# Macrolide and fluoroquinolone associated mutations *in Mycoplasma genitalium* in a retrospective study of male and female patients seeking care at a STI Clinic in Guangzhou, China, 2016-2018

**DOI:** 10.1186/s12879-020-05659-3

**Published:** 2020-12-11

**Authors:** Wujian Ke, Dongling Li, Lai Sze Tso, Ran Wei, Yinyuan Lan, Zhengyu Chen, Xiaohui Zhang, Liuyuan Wang, Chunmei Liang, Yuying Liao, Huiru Chen, Yahui Liu, Heping Zheng, Ligang Yang

**Affiliations:** 1 Department of Sexually Transmitted Diseases, Dermatology Hospital, Southern Medical University, Guangzhou, 510095 China; 2 Department of Sexually Transmitted Diseases, Guangdong Provincial Dermatology Hospital, Guangzhou, 510095 China; 3 Department of Culture Studies and Oriental Languages, University of Oslo, 0315 Oslo, Norway; 4 Anthropology, Massachusetts Institute of Technology, Cambridge, MA 02142 USA; 5 Center for Health and Human Development Studies, Sun Yat-Sen University, Guangzhou, 510275 China; 6 Department of Dermatovenerology, Tianjin Medical University General Hospital, Tianjin, 300052 China; 7 Clinical Laboratory, Dermatology Hospital, Southern Medical University, Guangzhou, 510095 China; 8 Department of Dermatology, Qingyuan Chronic Disease Prevention Hospital, Qingyuan, 511500 China; 9 Dermatology Hospital, Southern Medical University, Guangzhou, 510091 Guangdong China

**Keywords:** Antimicrobial resistance, Macrolide, Fluoroquinolone, Mutations, Sexually transmitted infection, *Mycoplasma genitalium*, 23S rRNA, *gyrA*, *parC*, Guangzhou, China

## Abstract

**Background:**

Antimicrobial resistance in *M. genitalium* is a growing clinical problem. We investigated the mutations associated with macrolide and fluoroquinolone resistance, two commonly used medical regimens for treatment in China. Our aim is to analyze the prevalence and diversity of mutations among *M. genitalium*-positive clinical specimens in Guangzhou, south China.

**Methods:**

A total of 154 stored *M. genitalium* positive specimens from men and women attending a STI clinic were tested for macrolide and fluoroquinolone mutations. *M. genitalium* was detected via TaqMan MGB real-time PCR. Mutations associated with macrolide resistance were detected using primers targeting region V of the 23S rRNA gene. Fluoroquinolone resistant mutations were screened via primers targeting topoisomerase IV (*parC*) and DNA gyrase (*gyrA*).

**Results:**

98.7% (152/154), 95.5% (147/154) and 90.3% (139/154) of *M. genitalium* positive samples produced sufficient amplicon for detecting resistance mutations in 23S rRNA, *gyrA* and *parC* genes, respectively. 66.4% (101/152), 0.7% (1/147) and 77.7% (108/139) samples manifested mutations in 23S rRNA, *gyrA* and *parC* genes, respectively. A2072G (59/101, 58.4%) and S83I (79/108, 73.1%) were highly predominating in 23S rRNA and *parC* genes, respectively. Two samples had amino acid substitutions in *gyrA* (M95I and A96T, respectively). Two samples had two amino acid substitutions in *parC* (S83I + D87Y). 48.6% (67/138) of samples harbored both macrolide and fluoroquinolone resistance-associated mutations. The most common combination of mutations was A2072G (23S rRNA) and S83I (*parC*) (40/67, 59.7%). One sample had three amino acid changes in 23S rRNA, *gyrA* and *parC* genes (A2072G + A96T + S83I).

**Conclusions:**

The high antimicrobial resistance rate of *M. genitalium* in Guangzhou is a very worrying problem and suggests that antimicrobial resistance testing and the development of new antibiotic regimens are crucially needed.

## Background

Antimicrobial resistance (AMR) of *Mycoplasma genitalium* (*M. genitalium*) is a growing problem with global implications for clinical guidelines and treatment [1–6]. As Jensen and Bradshaw (2015) argue, clinical monitoring and effective reporting on antimicrobial resistance-mediating mutations in *M. genitalium* across geographic regions and populations are crucial for developing effective treatments in managing *M. genitalium* infections and AMR-mediation across global settings [7]. Yet, despite being one of the most populous countries in the world, there is sparse data on the prevalence of AMR-related mutations in China. Here, we contribute to global efforts to address this gap in AMR surveillance. We investigate the rates of AMR mutations associated with macrolide and fluoroquinolone treatment failure in *M. genitalium*, expanding the discussion on the use of these antibiotics in Guangzhou, China. Given these alarmingly high rates, it is very important to understand the background of antibiotic use in this region.

*M. genitalium* can result in urethritis [8], Mucopurulent cervicitis (MPC) [9], endometritis [10], and pelvic inflammatory disease (PID) [11–13]. *M. genitalium* is also a suspected cause of reactive arthritis and proctitis [14]. Characteristics contributing to increased risk of *M. genitalium* infections include living in low-and-middle-income localities [15–18], experiencing fertility problems for both men [19, 20] and women [21], abnormal pregnancy status [22], and being members of vulnerable populations, including men-who-have-sex-with men (MSM) [23, 24], female sex workers (FSW) [25], and people living with HIV [26]. These considerations have spurred clinicians and public health agencies to call for global coordination of *M. genitalium* guidelines and treatment to help mitigate AMR-related problems [1–6].

Due to the lack of a cell wall, the treatment choice of *M. genitalium* was limited to tetracyclines, macrolides, or later-generation fluoroquinolones [7, 27]. According to the 2016 European NGU guidelines, patients with urethritis should be tested for *C. trachomatis* and *M. genitalium* via nucleic acid amplification testing [28]. Since a single-dose treatment of azithromycin may result in the development of antimicrobial resistance in *M. genitalium* [29], the 2015 UK NGU guidelines, the 2016 European *M. genitalium* guidelines and the Australian STI management guidelines (2018) no longer recommend azithromycin 1 g as first line therapy [30–32]. Hence, it is troubling that mounting evidence indicates *M. genitalium* drug-resistance increases with even just a single-dose treatment of azithromycin [2, 33–35]. Although a single dose of AZM can result in the development of resistance, AZM can be used in conjunction with doxycycline for susceptible infections (no 23S mutation, where screening is available). The fluoroquinolone moxifloxacin, another medication extensively used as a second-line bactericidal, has a cure rate approaching 100% in infections with susceptible strains [36]. In recent years, a decline in the efficacy of moxifloxacin has also been noted, first in Japan [7] then in Australia [37–39]. The elimination rate of moxifloxacin for *M. genitalium* infection has decreased from 100 to 89% since 2010 [37].

Genetically, mutations resulting in macrolide resistance are primarily attributed to single-nucleotide polymorphism (SNP) at positions A2071 or A2072 in region V of the 23S rRNA gene [2, 40]. Fluoroquinolone resistance is attributed to alternations of the *gyrA* subunit in DNA gyrase (which is composed of two *gyrA* and two *gyrB* subunits), or the *parC* subunit of topoisomerase IV (which is composed of two *parC* and two *parE* subunits) [41]. Compared with *parC* mutations, *gyrA* mutations likely have a less-severe effect on reducing the susceptibility of the bacterium for fluoroquinolone [42, 43]. Moxifloxacin resistant *M. genitalium* isolates, primarily causing amino acid changes at positions S83 and D87 (*M. genitalium* numbering) of *parC*, are similar to those found in other fluoroquinolone resistant bacteria [33, 44–46]. AMR studies of fluoroquinolone resistance in *M. genitalium* DNA tend to amplify the quinolone-resistance determining region (QRDR) of the *gyrA* gene and the corresponding region of the *parC* gene from *M. genitalium* DNA [47]. Antibiotic resistance of *M. genitalium* to both macrolides and quinolones have been found in Japan, Australia, and New Zealand since 2008 [33, 38, 44, 48, 49]. This disturbing trend suggests that the AMR dilemma attributable to *M. genitalium* is spreading and becoming even more virulent [33, 44, 48, 49].

Clinical monitoring and effective reporting of findings and research of antimicrobial resistance-mediating mutations in *M. genitalium* across geographic regions and populations are crucial for the development of efficacious treatment for combating *M. genitalium* infections and managing AMR across global settings [7]. Unfortunately, there is sparse data and low awareness of the patterns of antimicrobial resistance of *M. genitalium* in China. The aim of this study is to support and contribute to AMR research by analyzing the prevalence and diversity of mutations associated with macrolide and fluoroquinolone resistance among *M. genitalium* in positive clinical specimens in Guangzhou, China.

## Methods

### Study population and specimens

A total of 154 *M. genitalium* positive clinical specimens were collected from patients attending a STI clinic at Dermatology Hospital, Southern Medical University, Guangzhou, China. The collecting period was from December 2016 to December 2018. The samples included urethral swabs and/or rectal swabs from male patients and cervical swabs from female patients.

*M. genitalium* was detected via TaqMan MGB real-time polymerase chain reaction (PCR) as described by Jensen et al. [50]. DNA extracted by using DNA extraction kit (Suzhou Bacme Biotech Co.) from *M. genitalium* positive samples were tested for the mutations associated with macrolide and quinolone resistance in the same day or stored at − 20 °C prior to testing. Samples in our study were collected with the permission of STI patients as part of standard protocol for diagnostics and treatment. Specimens were then processed and stored with no identifiable patient data. As part of hospital protocol, de-identified patient samples are stored in the STI clinic biobank for surveillance, diagnosis, and research purposes. This study utilizes specimens from this biobank.

### Detection of macrolide and fluoroquinolone resistance-associated mutations in 23S rRNA, gyrA, and parC

From extracted DNA, mutations associated with macrolide resistance were detected using primers targeting region V of the 23S rRNA gene (nucleotides 1992–2138) [40]. Fluoroquinolone resistance mutations in the *gyrA* (nucleotides 172–402) and *parC* (nucleotides 164–483) genes were screened using primers as reported previously [51, 52]. Details of the primer sequencing and thermo-cycling parameters for amplification are provided in Table 1.

**Table 1:**
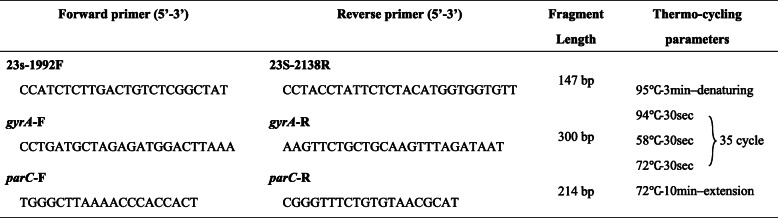
Primer sequences and thermo-cycling parameters for amplification of resistance-determining regions.

*bp* base pair

Each reaction volume of 25 μl contained 10 × PCR buffer (without Mg2+: 100 mM Tris-HCl pH 8.8 at 25 °C; 500 mM KCl, 0.8%(v/v) Nonidet), 0.6 μM each forward and reverse primer, 0.5 μl dNTP 10 mM, 1 U of P*fu* DNA polymerase (Invitrogen) and sterile water. Confirmation of PCR product was achieved by using an agarose gel (1.5%) electrophoresis. Amplified fragments were purified by QIAquick PCR Purification Kit (QIAGEN). Sequencing services were purchased and outsourced to Sangon Biotech, China. Samples were sequenced in both directions.

### Data analysis

The mutation sequencing data in 23S rRNA, *gyrA* and *parC* genes from *M. genitalium* positive DNA specimens were analyzed with the software program BioEdit (http://www.mbio.ncsu.edu/bioedit/bioedit.html). The genome sequence of *M. genitalium* strain G37 (GenBank accession no. NC_000908.2) was used as a reference strain. Amino acid changes in the QRDRs of the *gyrA* and *parC* genes in this study were compared with previous studies [6, 24, 26, 33, 38, 41].

### Ethics approval

The study was approved by the Ethics Committee of Dermatology Hospital of Southern Medical University (approval no. GDDHLS - 20,171,203, 13/12/2017). Informed consent was waived as the committee believes that this research presents no potential risk because the study does not contain identifiable data that would cause harm or result in a breach of confidentiality.

## Results

### Laboratory results of AMR mutations in M. genitalium

A total of 154 *M. genitalium* DNA-positive samples were stored during the collection period (December 2016–December 2018). Of these, 98.7% (152/154), 95.5% (147/154) and 90.3% (139/154) produced sufficient amplicon for detecting resistance mutations in 23S rRNA, *gyrA* and *parC* genes, respectively.

Results are summarized in Table 2.

**Table 2:** Prevalence of mutations associated to macrolide and fluoroquinolone resistance from 154 *M. genitalium* positive samples in Guangzhou, China, 2016–2018

Gene	SNP^**a**^	Amino acid change	Frequency, % (No. of samples containing mutation(s) or wild type / No. of successfully sequenced samples)
23S rRNA	A-2071 → G	-b	13.8 (21/152)
	A-2071 → T	–	13.2 (20/152)
	A-2072 → G	–	38.8 (59/152)
	A-2072 → C	–	0.7 (1/152)
	Wild type	–	33.6 (51/152)
*gyrA*	G-285 → C	M-95 → I	0.7 (1/147)
	G-286 → A	A-96 → T	0.7 (1/147)
	Wild type	–	98.6 (145/147)
*parC*	C-234 → T	No change	0.7 (1/139)
	G-241 → T	G-81 → T	0.7 (1/139)
	A-247 → C	S-83 → R	2.2 (3/139)
	G-248 → A	S-83 → N	5.8 (8/139)
	G-248 → T	S-83 → I	56.8 (79/139)
	T-249 → A	S-83 → R	1.4 (2/139)
	G-259 → T	D-87 → Y	2.9 (4/139)
	G-259 → A	D-87 → N	2.2 (3/139)
	A-260 → G	D-87 → G	2.9 (4/139)
	T-267 → C	No change	0.7 (1/139)
	G-248 → T **+** G-259 → T	S-83 → I **+** D-87 → Y	1.4 (2/139)
	Wild type	–	22.3 (31/139)

^a^ Nucleotide positions in 23S rRNA and in *gyrA* and *parC* genes are given according to the *M. genitalium* G37 genome (GenBank accession no. NC_000908.2). S*NP* single-nucleotide polymorphism, *rRNA* ribosomal ribonucleic acid, *b*, no amino acid change

Among 152 samples, 66.4% (101/152) harbored mutations in the 23S rRNA gene, and 33.6% were wild type. The mutation A2072G (*n* = 59) was highly predominating in Guangzhou, accounting for 58.4% (59/101) of the cases found positive for nucleotide substitutions in the 23S rRNA gene. Other detectable mutations included A2071G (*n* = 21), A2071T (*n* = 20), and A2072C (*n* = 1).

Although, none of the *gyrA* mutations have been associated with increased fluoroquinolone MICs, amino acid alterations in *gyrA* (M95I, A96T) were only detected in two samples. To our knowledge, this specific amino acid change (A96T) has not been reported elsewhere in the AMR literature. However, mutation at the next position (99 in *gyrA*) have been described in previous reports, and the positions are within the QRDRs, indicating its association with fluoroquinolone resistance [45]. Mutations of *parC* at positions S83 (except S83N) and D87 have been associated with increased MICs of moxifloxacin and *parC* mutations typically associated with fluoroquinolone resistance were detected in 77.7% (108/139) of samples. Of these, the most frequent mutation was S83I (*n* = 79), accounting for 73.1% of 108 samples. In two samples, two amino acid substitutions in *parC* (S83I + D87Y) were present. As shown in Table 2, substitutions in *parC* are more common than in *gyrA*.

Of the 138 samples undergoing complete analysis for both the 23 s RNA and *parC* genes, 48.6% (67/138) harbored both macrolide and fluoroquinolone resistance-associated mutations. Combining the 23S rRNA and *parC* mutations, 15 genotypes were identified (Table 3).

**Table 3:** Prevalence of 23S rRNA gene and *parC* mutations among *M. genitalium* positive samples with possible multidrug resistance in Guangzhou, China, 2016–2018

Mutations^**a**^	Count (%)^**b**^
A2071G + S83I	8 (5.8)
A2071G + S83R	2 (1.4)
A2071G + S83N	1 (0.7)
A2071G + D87G	1 (0.7)
A2071G + D87N	1 (0.7)
A2071T + S83I	1 (0.7)
A2071T + S83R	2 (1.4)
A2071T + D87N	1 (0.7)
A2071T + D87Y	1 (0.7)
A2072G + G81C	1 (0.7)
A2072G + S83I	40 (29.0)
A2072G + S83R	3 (2.2)
A2072G + S83N	1 (0.7)
A2072G + D87Y	3 (2.2)
A2072G + S83I + D87Y	1 (0.7)

^a^ Nucleotide positions in 23S rRNA and in *parC* gene are listed in accordance to the *M. genitalium* G37 genome (GenBank accession no. NC_000908.2). ^b^ Only 138 successfully sequenced samples included

## Discussion

The mutations in 23S rRNA and *parC* have been identified as the cause of failure of macrolide and quinolone in the treatment of *M. genitalium* [53, 54]. In our study, 66.4% (101/152) and 77.7% (108/139) of samples manifested mutations in 23S rRNA and *parC* genes, and A2072G (59/101, 58.4%) and S83I (79/108, 73.1%) were highly predominating in 23S rRNA and *parC* genes, respectively. More worryingly, the proportion of mutations in both 23S rRNA and *parC* genes was as high as 48.6%. This suggests that nearly half of these samples are resistant to both macrolide and quinolones. We also reported other mutations in *parC* and *gyrA* genes. However, the significance of these mutations requires further study.

Within the last 10 years, *M. genitalium* eradication rate has declined gradually [55, 56]. The resistance rate of *M. genitalium* has been described as a rising phenomenon in many countries [42, 57]. At the time of this study, there are only three locations actively conducting AMR-related research in clinical settings in China. The earliest published macrolide-associated mutations *in M.genitalium* in China collected samples from 18 symptomatic NGU patients [58]. In this research, the 23S rRNA mutation rate was 94.4%, with A2072G being the most common (55.6%), A2071G the second most (27.8%), and A2071T as the third most common mutation (11.1%), with no double-mutations detectable [58]. Later, in the same hospital, 358 *M. genitalium* positive samples were collected. The 23S rRNA mutation rate was 88.9%, with A2072G being the most common (61.9%), A2071G the second most (17.6%), and no double-mutations were detected [59]. The *parC* mutation rate was 90.4%, S83 → I was the most common mutation (83.7%) [59]. The double mutation in *parC* (G248A + G259T) was detected [59]. The *gyrA* mutation rate was 13.0%, with M95 → I being the most common (5.3%), three double-mutations in G244A + G285A, G285A + A309G, and G285A + A317G were detectable [59]. Another earlier study collected samples among men seeking care at an infertility clinic in Changsha, a city in the interior of China [60]. The macrolide mutations rate was similarly extremely high at 96.7% [60]. The two most common mutations in the Nanjing study are also the most frequent mutations in Changsha, that is, A2072G (60.0%) and A2071G (20.0%) [60]. Unlike in Nanjing, the analysis conducted on specimens from Changsha detected double-mutations, and these mutations are frequent enough to be the third most common set of mutations (A2071T + A2072G at 11.7%). Our location, a STI center based in a hospital in Guangzhou, constitutes the third AMR site. Our facility is a provincial STI center situated in Guangzhou, the capital city of Guangdong Province. Guangzhou is an international hub for travel, trade, and commerce and a major destination for migrants and their concomitant illnesses. Servicing the medical needs of such a diverse population, we focus on macrolide and fluoroquinolone resistance-associated mutations in *M. genitalium.* We extend current knowledge in two key ways. First, we continue monitoring and reporting efforts on macrolide and fluoroquinolone resistance, expanding on reports from the two prior studies based in central and interior China, by adding a major urban migration destination in south China. Second, we expand on AMR surveillance by being the first to report on macrolide and fluoroquinolone-associated mutations in men and women in China.

The 23S rRNA mutation is associated with macrolide resistance [61]. We found that SNPs in region V of the 23S rRNA gene were observed in 101 (66.4%) samples from male and female patients with *M. genitalium*-positive infection in 2016–2018. Mutations mainly occurred at positions A2071 and A2072 mainly to G (C or T is relatively less). With the exception of a study from Greenland, the mutation frequency (66.4%) [62] observed was higher than frequencies reported by Russia and Estonia (0.7 ~ 10%) [63], South Africa (10%) [64], southern Sweden (13%) [65], France (17%) [66], Japan (42.2%) [42], southern USA (48%) [67], Norway (56%) [68], and Denmark (57%) [68]. However, our rate of 66.4% is lower than rates reported from England (82.4%) [69], the US (Alabama: 74.1% HIV positive MSM) [26], and Australia (79.4%) [70].

It is widely reported that *M. genitalium* expressed a diversity of mutations linked to fluoroquinolone resistance-associated in *gyrA* and *parC* gene [49, 53, 68]. Similar to extant studies, mutations in the QRDR of the *gyrA* gene of our samples were rarely detected [53, 71]. The amino acid changes (M95 → I and A96 → T) in *gyrA* were found in our specimens. The M to I transition at position 95 of *gyrA* (G to C at nucleotide position 285) was first reported in 2013 by Tagg et al. [49], most commonly observed from 2013 to 2017 in Japan, and have been reported in moxifloxacin-resistant strains of *M. pneumoniae*, *M. hominis*, and *Ureaplasma spp* [49, 57, 72, 73]. To our knowledge, a *gyrA* A96 → T mutation in the core of the QRDR has not previously been described in *M. genitalium* and its association with resistance to fluoroquinolone remains unknown. The amino acid changes at G81, S83 and D87, have been previously reported as being associated with fluoroquinolone resistance in *M. genitalium* and other closely related organisms [44, 46, 49]. Although the majority of published reports have shown the *parC* S83N and S83I substitution as the two most prevalent base changes at position 248, we find that the S83I substitution accounted for 71.8% (79/110), significantly higher than reports from Japan (13.0–23.2%) [42], New Zealand (16.7%) [48], and southwestern France (9.1%) [66]. Among the 139 samples successfully amplified DNA sequences of *parC* gene, we observed an exorbitantly high mutation rate of 77.7%.

Additionally, 48.6% (67/138) of samples were multidrug resistant and contained both macrolide and fluoroquinolone resistance related SNPs. If SNP on *parC* is strictly limited to S83I, the multidrug resistance rate was 36.1% (50/138). In Japan, the prevalence of multidrug resistance with A2071G or A2072G in the 23S rRNA and amino-acid change in S83 or D87 of *parC* has been reported in up to 21.8% from 2010 to 2017 [42]. Our data showed very high prevalence (47.8%) of the same mutation. This trend of multidrug resistance presents challenges for clinicians because of a lack of suitable alternative therapy after azithromycin and moxifloxacin failure. Pristinamycin as the only third-line treatment has been reported to be only about 75% effective and is not readily available in China [27].

The high prevalence of mutations in macrolide and quinolone resistance-associated genes observed in our study might be related to the study population and to antibiotic overuse in China. The clinical samples were collected from the STI clinic of Dermatology Hospital, Southern Medical University. As a provincial level STI center, our doctors are referred patients from all over the region when doctors from feeder hospitals are unable to resolve medical ailments locally. These patients likely experienced several prior courses of antibiotic treatment. In addition, in China, it is incredibly easy for the public to obtain antibiotic prescriptions and purchase antibiotics in pharmacies. Data show that antibiotic use in children and hospitalized patients in China is very high [74]. These factors further exacerbate the problems of antibiotics resistance confronting health facilities today [75].

### Limitations

An important limitation of the study is the lack of epidemiological and clinical information, as well as information about treatment received and clinical evolution of the patients. The significance of several novel mutations in the *parC* and *gyrA* genes remains unknown. Nonetheless, the prevalence of mutations associated to macrolide and fluoroquinolone resistance in our study related to phenotypic testing has been previously reported in several studies. Our prevalence rate is a calculation based on a sample of patients seen by clinicians at our STI clinic. During patient intake and consultation, we did not collect patients’ history of previous antibiotic use. We hypothesized that patients at our STI clinic were more likely to have previously used antibiotics than the general population, so there might be a possibility of overestimating the prevalence rate when extended to the general population. Secondarily, we lack data for a large sample epidemiological survey of *M. genitalium,* since samples studied were collected mainly from a single clinic. Hence, our findings might not be representative or readily generalizable to the larger population living in Guangzhou.

## Conclusions

In conclusion, the high mutation rate of *M. genitalium* reported in this study is a very worrying problem. For patients with *M. genitalium* infection, antimicrobial resistance testing is crucial. The occurrence of drug-resistant strains is of great public health concern. The development of new antibiotic regimens for *M. genitalium* infections are urgently needed.

## Data Availability

The datasets generated and/or analysed during the current study are available in the GenBank repository, accession number to datasets are BankIt2402413, BankIt2402439 and BankIt2402448.

## Abbreviations

AMR: antimicrobial resistance
DNA: Deoxyribonucleic acid
FSW: Female sex worker
HIV: Human immunodeficiency virus
MPC: *Mucopurulent cervicitis*
*M. genitalium*: *Mycoplasma genitalium*
*M. hominis*: *Mycoplasma hominis*
*M. pneumonia*: *Mycoplasma pneumonia*
*MSM*: Men who have sex with men
NAAT: Nucleic acid amplification testing
TaqMan MGB: TaqMan minor groove binder
PCR: Polymerase Chain Reaction
PID: Pelvic inflammatory disease
QRDRs: Quinolone resistance-determining regions
rRNA: Ribosomal ribonucleic acid
SNPs: Single nucleotide polymorphisms
STI: Sexually Transmitted Infection
